# Characterisation of a flavonoid ligand of the fungal protein Alt a 1

**DOI:** 10.1038/srep33468

**Published:** 2016-09-16

**Authors:** María Garrido-Arandia, Javier Silva-Navas, Carmen Ramírez-Castillejo, Nuria Cubells-Baeza, Cristina Gómez-Casado, Domingo Barber, Juan C. Pozo, Pablo G. Melendi, Luis F. Pacios, Araceli Díaz-Perales

**Affiliations:** 1 Centre for Plant Biotechnology and Genomics (UPM-INIA), Pozuelo de Alarcón, Madrid, Spain; 2 Lund University, Department of Experimental Medical Sciences, Lund, Sweden; 3 Institute of Applied Molecular Medicine (IMMA), CEU San Pablo University, Spain; 4 Department of Natural Systems and Resources, ETSI Montes, Technical University of Madrid, Madrid, Spain

## Abstract

Spores of pathogenic fungi are virtually ubiquitous and cause human disease and severe losses in crops. The endophytic fungi *Alternaria* species produce host-selective phytotoxins. Alt a 1 is a strongly allergenic protein found in *A. alternata* that causes severe asthma. Despite the well-established pathogenicity of Alt a 1, the molecular mechanisms underlying its action and physiological function remain largely unknown. To gain insight into the role played by this protein in the pathogenicity of the fungus, we studied production of Alt a 1 and its activity in spores. We found that Alt a 1 accumulates inside spores and that its release with a ligand is pH-dependent, with optimum production in the 5.0–6.5 interval. The Alt a 1 ligand was identified as a methylated flavonoid that inhibits plant root growth and detoxifies reactive oxygen species. We also found that Alt a 1 changes its oligomerization state depending on the pH of the surrounding medium and that these changes facilitate the release of the ligand. Based on these results, we propose that release of Alt a 1 should be a pathogenic target in approaches used to block plant defenses and consequently to favor fungal entry into the plant.

*Alternaria* is a genus of endophytic fungi that includes more than 50 species, many of which are plant pathogens. In humans, this species is responsible for several types of infection and disease in humans, such as hypersensitivity, pneumonitis, bronchial asthma, allergic sinusitis, and rhinitis^1^,^2^,^3^,^4^. *Alternaria* spp. are found throughout the world, particularly in warm regions, where their spores are present in the atmosphere throughout the year, with peaks in spring, summer, and autumn^5^.

*A. alternata* infects more than 100 plant species. Its pathogenicity can be attributed to the production of diverse host-selective phytotoxins or bioactive host-dependent metabolites, as well as to increased production of toxic reactive oxygen species (ROS) involved in cell death^6^. Since *Alternaria* fungi are endophytic, they can remain on the surface of the host plant without inducing symptoms while waiting for the right moment to germinate. However, the molecular mechanisms underlying the interactions between endophytic fungi and their host plants remain largely unexplored^7^.

Fungal flavonoid-type compounds have been reported to play a role in plant defense against fungi^8^,^9^. Flavonoids are well-known secondary plant metabolites involved in a variety of processes such as cell signaling, plant growth, and reproduction^10^,^11^,^12^,^13^,^14^. Among this heterogeneous group of molecules, flavone derivatives with two *ortho*-hydroxyl groups on the B-ring (catechol) are particularly interesting owing to their capacity to detoxify ROS^12^,^15^,^16^,^17^ and activate siderophores^18^,^19^; both features are relevant in plant defense strategies.

The allergen Alt a 1 (AAM90320.1, NCBI Protein Database) is detected in the spores of *A. alternata* before germination^20^,^21^. It has been described as the main allergen associated with chronic asthma^22^ and was recently shown to interact with the pathogenesis-related plant defense protein PR5^23^, although the molecular mechanism of Alt a 1 in fungal pathogenesis remains unknown^24^. Alt a 1 is coded by a unique gene (AHA82637.1) that is present only in *Alternaria* and related species^25^. The expression of this gene in *Arabidopsis thaliana* has been related to infection^26^. Alt a 1 is a 30-kDa homodimer that dissociates into two subunits of 14.5–16 kDa under reducing conditions^27^. The monomeric crystal structure comprises a β-barrel composed of 11 β-strands that is a unique type of fold with no equivalent in the Protein Data Bank^7^.

Herein, we report new results that shed light on the versatility of this singular protein. We show that Alt a 1 is not secreted in its apo-form (without ligand), but that it transports a flavonoid compound into host cells. We also demonstrate that the Alt a 1 oligomerization state changes depending on both the presence of its ligand and the pH of the surrounding medium. The ligand, which was stained with diphenyl boric acid 2-aminoethyl ester (DPBA), inhibits plant cell growth and exhibits antioxidant activity. Although we did not fully elucidate the identity of the ligand, we were able to establish that it was a flavonoid. Based on these data, we suggest that the ligand of Alt a 1 is involved in ROS detoxification as a defense mechanism in fungi such as *Alternaria*.

## Results

### The Alt a 1–flavonoid complex is released in a pH-dependent manner

**Figure 1:**
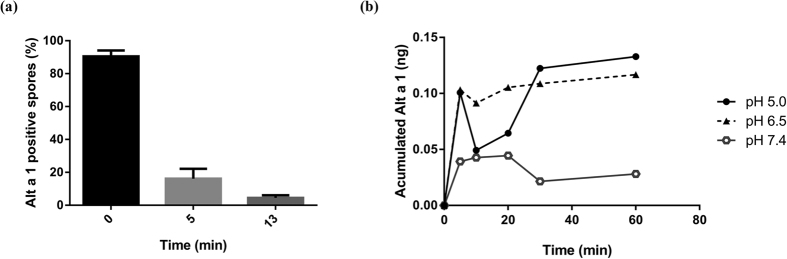
Alt a 1 protein is located inside *A. alternata* spores. **(a)** Spores containing Alt a 1 were quantified at different times and represented as a percentage of Alt a 1-positive spores. **(b)** Alt a 1 release is pH-dependent. Spores (10^6^) were incubated at different pHs, and the quantity of Alt a 1 protein was quantified by ELISA using a specific polyclonal antibody.

Using confocal microscopy, Alt a 1 was located inside spores of *Alternaria* by immunofluorescence with specific monoclonal antibody; the most intense signal was observed in the spore cytoplasm at time 0 (before spores were placed in water media). This signal decreased with time, indicating that Alt a 1 is already preformed in the spore and is then secreted when humidity is detected (Fig. 1a). Release of Alt a 1 is strongly pH-dependent and accumulates mainly in the first few minutes at pH values between 5.0 and 6.5 (Fig. 1b).

Alt a 1 was secreted with a phenolic compound, as confirmed by thin layer chromatography of the retained fraction using ethanol:formic acid:water (50:30:20) as eluent. DPBA staining revealed a spot that suggests the flavonoid nature of that ligand (data not shown)^11^.

**Figure 2:**
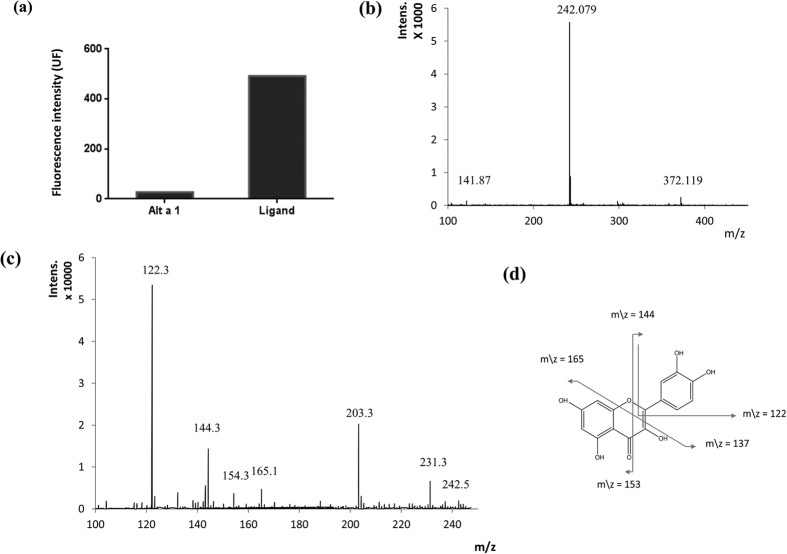
Alt a 1 is released transporting a flavonoid ligand. **(a)** The ligand (0.06 μg/μl) purified from Alt a 1 was incubated with DPBA (specific flavonoid staining, 0.25% w/v). Fluorescence intensity was measured in the TECAN GeniosPro system (λ_exc_ = 485 nm, λ_em_ = 535 nm). **(b)** MS-MALDI TOF analysis of the ligand. (**c)** MS/MS product ion mass spectrum of the ligand. **(d)** Fragmentation of quercetin^30^.

To characterize its chemical composition, the ligand was purified after ethanol extraction (70%) from a spore-mycelium mixture at room temperature, followed by high-performance liquid chromatography (HPLC). Peaks were stained with DPBA (Fig. 2a) and positive fractions were analysed using mass spectrometry (Fig. 2b). Three peaks were observed (m/z values 141.870, 242.079, and 372.119) and the MS/MS experiments revealed the presence of fragment ions with m/z values of 122.3, 144.3, 154.3, 165.1, 203.3, 231.3, and 242.5 (Fig. 2c). For comparison, the four lower mass peaks can be also found in fragmentation spectra obtained for quercetin according to the reported fragmentation pattern shown in Fig. 2d. This result indicates the presence of a flavonol skeleton in the ligand. The molecular mass of the ligand, 372.119 Da (Fig. 2b), corresponds to the quercetin value (302.2 Da) plus five methyl group (15 Da) substituents (see below). The two remaining peaks with higher mass in Fig. 2c are the result of initial fragmentation of the 372.119 peak.

### Alt a 1 is able to bind flavonoids with high affinity

**Figure 3:**
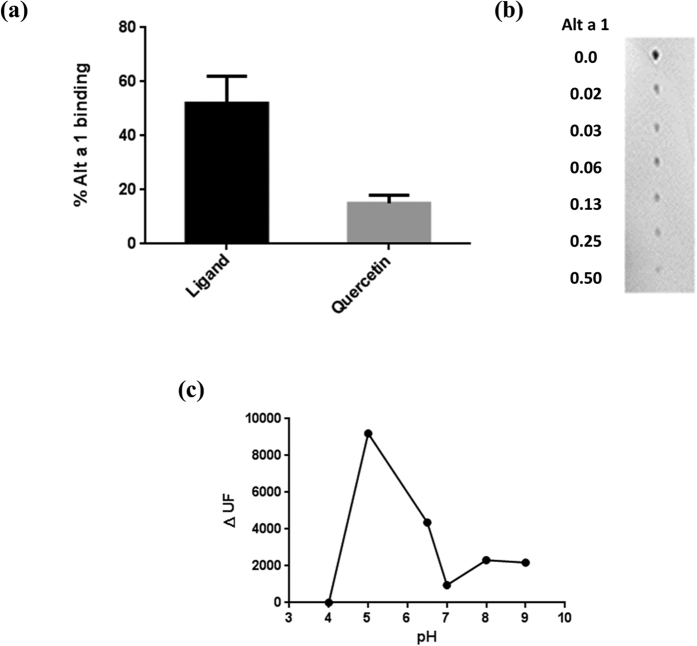
Alt a 1 binds its ligand in a concentration-dependent manner. **(a)** Dynabeads M-280 Tosylactivated were coated with Alt a 1 and incubated with its ligand. The percentage of Alt a 1-Dynabeads bound to the ligand is represented. **(b**) Increasing amounts of Alt a 1 (0.0–0.50 μg) were incubated with its ligand (1 μg) and stained with DPBA and dot-blotted onto nitrocellulose membrane. **(c)** Spores (10^6^) of *A. alternata* were incubated at different pHs for 1 hour at 25 °C. The presence of the ligand was detected with DPBA staining (0.25% w/v) in the TECAN GeniosPro system (λ_exc_ = 485 nm, λ_em_ = 535 nm). Quercetin was used as a flavonoid model.

Alt a 1 is able to bind quercetin (3,3′,4′,5,7-pentahy-droxyflavone, a well-known flavonol bearing a catechol moiety), as demonstrated by pull-down assays using Dynabeads^®^ M-280 Tosylactivated. The heated fraction was stained with DPBA (Fig. 3a). In contrast, no DPBA positive peak was observed before heating (data not shown), suggesting that fluorescence could be quenched when Alt a 1 is bound. A dissociation constant Kd = 0.7 μM was estimated for the Alt a 1-ligand complex (Fig. 3b). Release of the ligand from Alt a 1 was also strongly pH-dependent (Fig. 3c), with the maximum amount released at pH 5.0–6.5, which is the same interval as for Alt a 1 secretion.

### The flavonoid carried by Alt a 1 shows quercetin-like activity

**Figure 4:**
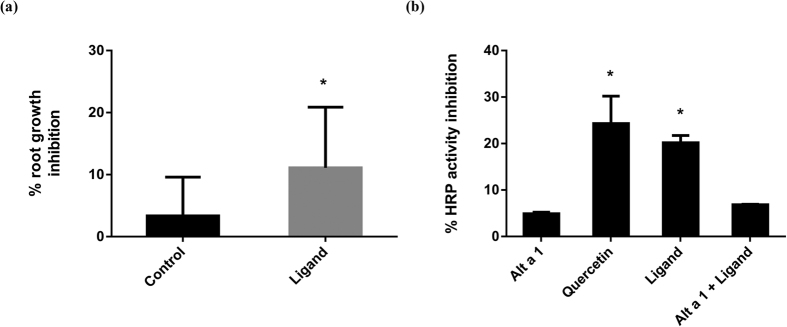
Effect of the ligand of Alt a 1 on *Arabidopsis thaliana* roots. **(a)** Root growth inhibition by treatment with the ligand of Alt a 1 measured as the percentage of inhibition with respect to root growth without ligand (n = 12). *p–values < 0.05 (*t* test). **(b)** Inhibition of HRP activity measured as percentage of inhibition with respect to total HRP activity (n = 5). *p–values < 0.05 (*t* test).

Quercetin and related polyhydroxylated flavones are known plant root inhibitors that also show ROS scavenger activity. Based on the results reported above, we studied both activities in the ligand of Alt a 1 protein. For the growth inhibition assays we used *Arabidopsis* root (Fig. 4a). Seeds were grown for six days and the ligand of Alt a 1 was then added to the medium. Root length was measured after 24 hours of treatment following the procedure previously described for quercetin^28^. The result (Fig. 4a) revealed that the ligand of Alt a 1 showed quercetin-like inhibition of root growth.

In order to study the ROS detoxification potential known for catechol-bearing flavonoids^29^, we tested the ability of the ligand of Alt a 1 to inhibit the activity of horseradish peroxidase (HRP) using ortho-phenylenediamine (OPD) as a substrate and hydrogen peroxide as a cofactor necessary for enzyme activity. The free ligand produced a significant increase in inhibition comparable to that of quercetin (Fig. 4b). When the HRP activity inhibition assay was performed with the ligand in the presence of Alt a 1, only a slight variation was observed (Fig. 4b). This result suggests that the chemical groups involved in antioxidant activity (catechol moiety in quercetin-like compounds) would be blocked by interactions with amino acid residues at the binding site of the protein.

### The flavonoid carried by Alt a 1 is modelled as a catechol-containing methylated flavonol

**Figure 5:**
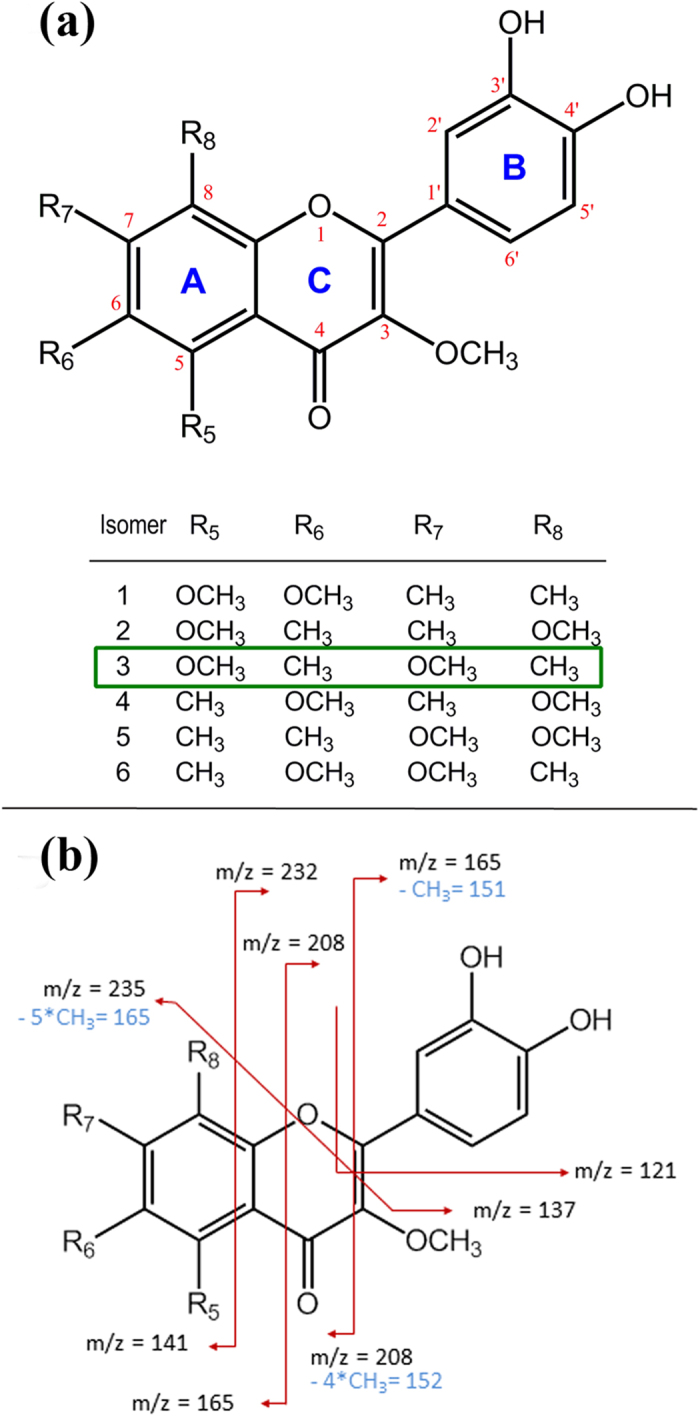
Proposed structures for the ligand of Alt a 1 and for the protein-ligand complex. **(a)** Structural formula of the flavone compound with the chemical composition C_20_H_20_O_7_ (molecular weight 372) proposed for the ligand of Alt a 1. The catechol moiety (B-ring) of quercetin involved in plant root growth inhibition and ROS detoxification is preserved. Six positional isomers for methyl groups in the A-ring are compatible with this proposal. Isomer 3 is the only structure with the five hydroxyl groups at the same positions as quercetin (5, 7 in A-ring). **(b)** Proposed fragmentation of the ligand of Alt a 1 based on the reported fragmentation of quercetin^30^ modified by the presence of methyl substituents (values in blue).

Based on the molecular mass, the similarity between the fragmentation profiles of flavonoids, the known existence of methylated flavones in fungi (see below), and the experimental evidence on flavonoid activity reported above, we propose the flavone-based structure shown in Fig. 5a as the ligand of Alt a 1. Its chemical composition is C_20_H_20_O_7_ (molecular mass 372), and the catechol moiety is preserved. There are six possible positional isomers compatible with this proposal although only one has the five hydroxyl oxygens at the same positions as quercetin (isomer 3 in Fig. 5a). The molecular mass of the ligand of Alt a 1, 372.119 Da (Fig. 2b) matches the quercetin value (302.2 Da) plus five methyl group substituents (15 Da). Based on the reported fragmentation of quercetin^30^ (Fig. 2d), the scheme in Fig. 5b depicts the putative analogous fragmentation for methylated derivatives of the structure in Fig. 5a. The resulting m/z values would correspond to the four lower mass peaks (122.3, 144.3, 154.3, and 165.1) in Fig. 2c.

### Docking of the modelled ligand to Alt a 1 indicates a tight protein–ligand interaction

**Figure 6:**
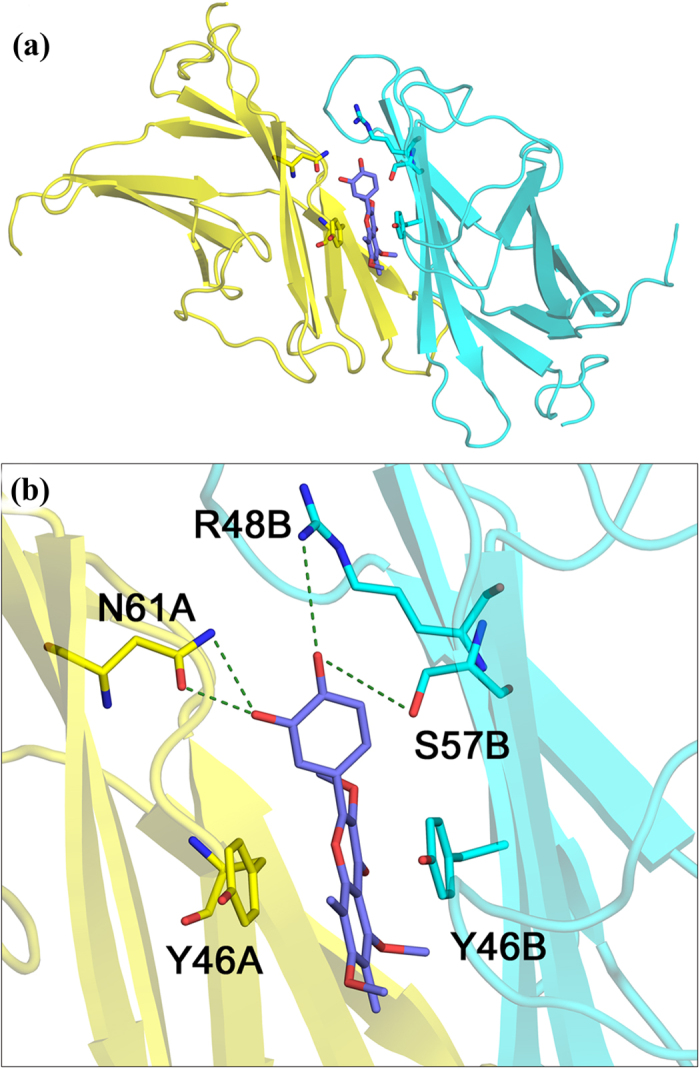
Model structure of the Alt a 1-ligand complex. **(a)** Structure of the recently identified inter-monomer β-barrel binding site of Alt a 1^31^ with isomer 3 of the ligand shown as sticks with carbons in deep blue at the geometry obtained in docking calculations. **(b)** Close-up of the binding site in (**a**) showing the main Alt a 1 residues involved in binding. Aromatic rings from two tyrosines in different subunits generate π-stacking with A- and C-rings of the ligand. One basic and two polar residues form hydrogen bonds (dashed green lines) with the OH groups of catechol.

Docking calculations at a grid search space enclosing the recently reported binding site of Alt a 1^31^ and using for the ligand a model 3D structure of isomer 3 (Fig. 5a) predict a geometry rather similar to that found for the Alt a 1-quercetin complex. The ligand is inside a β-barrel site composed of β-strands from two different protein chains in tetrameric Alt a 1 (Fig. 6a) and is located between two nearby Tyr residues from different subunits whose aromatic side chains form a π-stacked arrangement with the A- and C-rings of the ligand (Fig. 6a,b). Catechol (B-ring) interacts with three polar/charged residues from the two subunits that make hydrogen bonds with the two hydroxyl groups (Fig. 6b). For this complex, the docking results predict a protein-ligand binding free energy of −10.0 kcal/mol, i.e., a Kd of about 0.05 μM.

Docking calculations for the remaining positional isomers of the ligand (Fig. 5a) predict virtually the same geometries with binding free energies between −9.2 and −9.6 kcal/mol, i.e., Kd values between 0.2 and 0.09 μM. It is worth noting that the ligand structure with the lowest affinity energy (tightest binding) is isomer 3, which has the five hydroxyls at the same positions as quercetin. In all isomers, the catechol is strongly bound by a hydrogen bond between its two OH groups and amino acid side chains at the binding site. This result lends support to the suggestion above regarding blocking of catechol indicated by the considerable decrease in the antioxidant activity of the ligand in the presence of Alt a 1.

## Discussion

*Alternaria* species take several forms, ranging from saprophytes to endophytes to pathogens^6^. They are highly successful as fungal pathogens that cause severe losses in a wide variety of economically important crops and in many ornamental and weed species. The secretion of effector protein Alt a 1 before germination could be directly responsible for this success.

We showed that Alt a 1 is released quickly (during the first minutes), when spores detect humidity and that it is very sensitive to the pH of the surrounding medium^32^. The maximum release of Alt a 1 is observed at pH values between 5.0 and 6.5. This result is consistent with previous reports describing an optimum pH of about 6.0–6.5 for spore germination in *Alternaria*. In contrast, an optimum pH of about 4.0–4.5 was found for release of mycotoxins^33^. These pH values are typical of the apoplasts in ripe fruit^34^, where the spore is deposited and secretion takes place.

Alt a 1 is not released in an apo-form but carries a flavonol ligand with properties similar to those of quercetin, a plant flavonol model^35^. Protein-ligand affinity is high (Kd = 0.7 μM), and the presence of the ligand was found to be necessary for higher-order oligomerization, although still in a pH-dependent manner. In fact, the tetrameric form of Alt a 1, which carries the ligand, is stable at pH 6.5, whereas at pH 5.0 the tetramer breaks down^31^ to form monomers that release the flavonoid ligand to the medium.

Ligand transport has been described for many allergens, such as peach (Pru p 3), strawberry (Fra a 1), and birch pollen (Bet v 1)^36^,^37^,^38^. Although our results strongly support that the ligand of Alt a 1 is a flavonoid, its molecular mass does not match any similar compound in databases. However, the partial match of the quercetin fragmentation spectrum with that obtained for the ligand of Alt a 1 and the fact that its molecular mass corresponds exactly to that of quercetin plus five methyl substitutions provide essential clues in the identification of this ligand as a fungal methoxyflavonol. Although many methylated flavonols are known, their properties have not been determined, and—to an even lesser extent—their mass spectrometry data have not been recorded^39^,^40^. Besides, we still know relatively little about the presence of flavonoids in fungi. Some strains of *Aspergillus candidus* produce chlorflavonin, a methylated flavone^41^, and the presence of other flavones with several methyl and methoxy groups was detected in *Colletotrichum dematium*^9^. Most metabolites in fungi exhibit antifungal activity towards phylogenetically unrelated species, and the toxicity of flavones in this process has been found to decrease with the presence of methoxy groups^42^,^43^.

Based on the reported presence of methylated flavones and flavonols in fungi and, more particularly, similar fragmentation profiles of quercetin, together with plant root growth inhibition and ROS detoxification reported here, we hypothesize that the ligand of Alt a 1 is a molecule with the chemical composition C_20_H_20_O_7_ (molecular mass 372) and a flavone-based structural formula. Although there are several positional isomers compatible with this proposal, the framework structure we present fulfils two key conditions: (i) the catechol moiety (B-ring) is that of quercetin and is thus able to inhibit plant root growth and perform ROS scavenger activities when the ligand is not bound to the protein; and (ii) the structure incorporates methoxy and methyl substituents found in fungal flavones. Further studies will be performed to elucidate which of the six positional isomers is the actual structure of the ligand of Alt a 1.

In summary, we propose the following scenario for the versatility of Alt a 1. When *Alternaria* species germinates, the infected plant expresses pathogenesis-related proteins and produces ROS as a defense response. Alt a 1 is released mainly as a tetramer carrying its flavonol ligand in such a way that the catechol ring is blocked by interactions with protein residues at the binding site. When the Alt a 1-ligand complex reaches the plant apoplast, where the mildly acidic pH 5.5 leads to its breakdown, monomers and the ligand are released. In monomeric form, Alt a 1 interacts with plant defense proteins such as PR5^23^, and the free flavonol is able to reduce ROS levels to facilitate infection.

## Methods

### Fungal growth

*Alternaria* spores were isolated from kiwifruits by scraping the surface and then cultured on potato dextrose agar (PDA; Difco Becton Dickinson and Company, Sparks, MD, USA) with cefotaxime (200 μg/ml) (Calbiochem, Merck KGaA, Darmstadt, Germany), as previously described^23^. After 8 days, *Alternaria* spores were recovered with sterile water and stored at −80 °C in 20% glycerol.

### Immunohistochemistry assays

Spores of *A. alternata* were fixed with 4% formaldehyde in PBS, pH 7.4, at 4 °C for 15 min. After washing with PBS, the spores were permeabilized by 30 freeze-thaw cycles. After additional washing with PBS, the spores were incubated overnight at 4 °C with specific monoclonal anti-Alt a 1 antibodies (1:50, Bial Aristegui, Bilbao, Spain) and revealed using anti-mouse Alexa 488-conjugated antibodies (Molecular Probes). The specimens were mounted with glycerol:PBS (1:1) and observed with a Leica TCS-SP8 confocal microscope using the 488-nm laser excitation.

### Production of Alt a 1 and its ligand

Spores of *A. alternata* (10^6^) were incubated for 1 hour at 25 °C in media with different pHs (5.0, 6.5, and 7.4) at several times (5, 10, 20, 30, and 60 min). The supernatants were coated using enzyme-linked immunosorbent assay (ELISA) plates for 2 hours at 37 °C. After blocking, plates were washed and incubated with a polyclonal anti-Alt a 1 antibody (1:1000, Bial Aristegui) and HRP-IgG antibodies (1:30 000, Sigma-Aldrich, Madrid, Spain).

The presence of the ligand of Alt a 1 was quantified in the supernatants by specific flavonoid staining with diphenylboric acid 2-aminoethyl ester (DPBA; 0.25% w/v)^11^, and fluorescence was read at 485/535 nm in a microplate reader (TECAN GeniosPro Spectrafluor Fluorometer; TECAN Group, Männedorf, Switzerland). The amount of ligand released was quantified using a standard curve of known amounts of quercetin (Sigma-Aldrich).

### Isolation of Alt a 1

Alt a 1 was purified from spores and a mycelium mixture of *A. alternata* (ALK-Abelló, Denmark). Briefly, Alt a 1 was purified from defatted mixture and extracted with PBS buffer (0.1 mM sodium phosphate pH 7.4, 1.5 M NaCl, 1:5 (w/v), 1 h at 4 °C). The extract was dialyzed against H_2_O, freeze-dried, and fractionated by anion-exchange chromatography on a Waters Accell Plus QMA Sep-Pak cartridge (Waters Corp, Milford, MA, USA). Elution was carried out with 20 mM ethanolamine, pH 9.0, and the retained material was then eluted with 0.75 mM NaCl in the same buffer (1 ml/min). The Alt a 1 enriched fraction was repurified by phase reverse-high-performance liquid chromatography (HPLC) on a Nucleosil 300-C4 column (7 × 250 mm; particle size 5 μm: Tecknokroma, Barcelona, Spain). Elution was performed with a linear gradient of acetonitrile in 0.1% trifluoroacetic acid (10% for 5 min and 10–100% over 150 min; 1 ml/min).

Purified Alt a 1 was quantified using the commercial bicinchoninic acid test (Pierce, Cheshire, UK), and purity was measured using sodium dodecyl sulfate polyacrylamide gel electrophoresis (SDS-PAGE), mass spectrophotometric analysis with a Biflex III Spectrometer (Bruker–Franzen Analytik, Bremen, Germany), and fingerprinting after tryptic digestion using standard methods.

### Isolation of the ligand carried by Alt a 1

The ligand was extracted with ethanol (70%) from the spore-mycelium mixture at room temperature. After centrifugation, the supernatant was separated by HPLC (Nucleosil 120  C18, 250 × 4 mm, Tecknokroma) with an acetonitrile gradient of 10–85% for 30 min at 0.5  ml/min. Spectra were recorded at 254 and 280 nm. Peaks were analysed using DPBA staining (0.25% w/v)^29^, and quercetin (0.01 μg/μl) was used as a positive control. Data were recorded using a fluorescence microplate reader (TECAN GeniosPro Spectrafluor Fluorometer) at 485/535 nm.

### Alt a 1-flavonoid binding assays

To test the capacity of Alt a 1 to bind flavonoids, the isolated ligand and quercetin (Sigma-Aldrich) were used as a model. Immunoprecipitation assays were performed using Dynabeads M-280 Tosylactivated (Invitrogen, Barcelona, Spain) conjugated with Alt a 1 (0.2 μg/μl) and incubated with quercetin and its ligand (0.01 μg/μl). The Dynabeads were conjugated following the manufacturer’s instructions. After intensive washes, Alt a 1-Dynabeads were heated for 10 min at 95 °C, and binding was confirmed by staining of the supernatant with DPBA.

To study the kinetics of Alt a 1-flavonoid binding, increasing amounts of the protein were incubated with constant amounts of quercetin and ligand (0.25 μg) and stained with the fluorescent dye DPBA (0.25% w/v). The resulting sample was spotted onto a nitrocellulose membrane washed with water and visualized using an Alexa Fluor 488 laser in a Bio-Rad Pharos FX^TM^ Plus Molecular Imager. Dot intensity was calculated using the software provided with the instrument. All tests were performed in three independent assays. In the dissociation constant (Kd) of the Alt a 1-flavonoid (F) complex (Kd = [Alt a 1] [F]/[Complex]), the protein concentration [Alt a 1] was known, free flavonoid [F] was calculated by extrapolating the fluorescence obtained to a standard curve constructed with increasing amounts of flavonoid, and the concentration of the bound system [Complex] corresponded to the difference between the initial flavonoid [IF] and free flavonoid [FF], which was measured as explained above.

### Mass spectrometry analysis of the ligand of Alt a 1

Equal volumes of the sample (in ethanol) and the matrix solution (10 mg/ml 2.5-dihydroxybenzoic acid in ethanol) were mixed for a few seconds, and then 1 μl of the mixture was spotted onto an 800-μm AnchorChip target (Bruker-Daltonics) and allowed to dry at room temperature. For the analysis of flavonoid without a matrix, flavonoid solutions (1 μl) were applied directly to the MALDI target.

MALDI experiments were performed on an Autoflex III MALDI-TOF-TOF instrument (Bruker Daltonics) with a smartbeam laser. Samples were analyzed in positive ion detection mode and delayed extraction reflector mode in the mass range between 100 and 1000 m/z. The experimental parameters used were as follows: pulsed ion extraction 250 ns, laser frequency 1000 Hz, ion source 1 of 19.0 kV, ion source 2 of 16.60 kV, lens 8.79 kV, reflector 1 of 21.0 kV, and reflector 2 of 9.77 kV. Typically, 1000 laser shots were summed into a single mass spectrum. Laser intensity was modified according to the threshold energy necessary for the ionization of the sample, and the sample was also evaluated using different laser intensities (25 to 90%).

### UHPLC-ESqTOF of the ligand of Alt a 1

The analysis was performed in a micrOTOF-Q II mass spectrometer (Bruker Daltonics) equipped with an electrospray ionization source in positive ion mode. The instrument settings were as follows: capillary voltage, 4500 V; capillary exit, 130 V; dry gas temperature, 180 °C; and dry gas flow, 4 l/min. Spectra were obtained in positive ion mode. The mass spectrometer was used to carry out two scans: a full-mass scan between 50 and 1000 m/z at a repetition rate of 2 Hz, and an MS-MS scan of the most abundant ions in the full-mass scan. Argon was used as the collision gas. Collision energy was ramped at between 5 and 25 eV. Mass calibration was performed using sodium format clusters (10-mM solution of NaOH in 50/50% v/v isopropanol/water containing 0.2% formic acid).

### Plant root growth inhibition assays

Assays with *Arabidopsis* were performed using the Columbia ecotype. All seedlings were sown under sterile conditions on vertically oriented 12-cm square plates containing half-strength Murashige and Skoog (MS1/2) with 0.05% MES, 1% sucrose, and 1% plant-agar (Duchefa Biochemie B.V.) under a 16-h light/8-h dark photoperiod at 21–23 °C. To determine the antimitotic activity of the ligand, seeds were cultivated in the D-Root system, and primary root length was determined as previously described^28^. Data were analyzed using the *t* test.

### ROS inhibition assays

ROS activity was inhibited following a method published elsewhere^29^, with minor modifications, using Alt a 1, the flavonoid ligand of Alt a 1, quercetin, and the Alt a 1-ligand complex. Horseradish peroxidase 5 ng/ml (Sigma-Aldrich) was incubated with 0.03 μg/μl of the flavonoids, hydrogen peroxide (50 mM), and the substrate OPD (Thermo Scientific, Rockford, IL, USA). Activity was measured at 492 nm after 30 min of reaction. The percentage of inhibition was calculated considering the value obtained for the sample without flavonoid as 100% activity. All tests were performed in triplicate.

### Statistical analyses

Statistical analyses were performed using SPSS 17.0 and Graph Pad 6. The *t* test, Kruskal-Wallis test, one-way ANOVA, and two-way ANOVA with corrections for multiple comparisons were used when applicable. p-values < 0.05 were considered significant for all assays.

### Docking calculations

The initial spatial structures of the six positional isomers in Fig. 5A were constructed with the Build Structure tools included in Chimera 1.10.2^44^ and then optimized in several cycles of steepest descent and conjugate gradient minimizations using the Amberff12SB force field^45^ implemented in Chimera. Since the two binding sites in tetrameric Alt a 1 are symmetry-related and present the same binding affinity energies^31^, docking calculations were performed on only one half of the tetramer. Protein and ligand preparation for docking and spatial grid selection were also performed with the Autodock Vina tools in Chimera, and Vina calculations^46^ were then carried out for the six Alt a 1-ligand complexes. In all cases, the solution with lowest affinity binding free energy was selected. The molecular graphics (Fig. 5) were prepared and rendered with PyMOL 1.8.0^47^.

## Additional Information

**How to cite this article**: Garrido-Arandia, M. *et al*. Characterisation of a flavonoid ligand of the fungal protein Alt a 1. *Sci. Rep.* **6**, 33468; doi: 10.1038/srep33468 (2016).
